# Diagnostic yield of array-CGH in children with suspected rare disease

**DOI:** 10.1016/j.jped.2025.101450

**Published:** 2025-10-17

**Authors:** M. Carla Criado-Muriel, Ramón Arroyo-Ruiz, Elena Marcos-Vadillo, María Justel-Rodriguez, Lydia Alcubilla-García, María Isidoro-García, Pablo Prieto-Matos

**Affiliations:** a University of Salamanca (USAL), GIR of Biomedicine of Rare Diseases, Salamanca, Spain; b Institute of Biomedical Research of Salamanca (IBSAL), Salamanca, Spain; c University Hospital of Salamanca, Department of Pediatrics, Salamanca, Spain; d University of Salamanca (USAL), Department of Biomedical and Diagnostics Science, Salamanca, Spain; e University Hospital of Salamanca, Department of Clinical Biochemistry, Salamanca, Spain; f Clinical Rare Diseases Reference Unit DiERCyL, Castilla y León, Spain; g University of Salamanca (USAL), Department of Medicine, Salamanca, Spain; h University of Málaga, Faculty of Medicine, Málaga, Spain

**Keywords:** Rare diseases, Copy number variation, Array comparative genomic hybridization, Pediatric neurology, Genetic diagnosis, Clinical genetics

## Abstract

**Objective:**

This study aims to analyze the diagnostic yield of aCGH in pediatric patients with suspected rare diseases, focusing on its diagnostic value and effectiveness depending on different clinical symptoms.

**Methods:**

This observational study analyzed 600 aCGH tests performed in a pediatric unit (2018–2022) for patients with suspected rare diseases. DNA was extracted from peripheral blood; aCGH resolution was adjusted to clinical features. CNVs were classified per international guidelines. Forty sociodemographic, clinical, and genetic variables were analyzed using IBM SPSS v.26.

**Results:**

Of the 600 patients analyzed, 543 were included in the final study. The median age was 4.7 years (IQR: 6.36 years), and 66.3% were male. Most referrals came from pediatric neurology (84.3%), and the most common clinical manifestations were altered phenotype (38.6%), autism spectrum disorder (ASD) (38.6%), dysmorphia (28.2%), global developmental delay (GDD) (27.1%), and intellectual disability (21.0%). Among 543 patients, 30.4% presented CNVs, with 12.4% identified as pathogenic and 18.1% as variants of uncertain significance. Diagnostic yield was 12.2%, with 66 conclusive results — 90.9% of which were pathogenic. CNVs were most frequently detected on chromosomes 15 and 16. The highest yield was observed in clinical features such as coordination problems (35.7%), learning disorders (28.6%), and microcephaly (22.6%).

**Conclusion:**

The diagnostic yield of aCGH in this study was 12.2%. The test demonstrated higher diagnostic value in patients with multiple clinical manifestations, highlighting the importance of aCGH as a first-line diagnostic tool for rare diseases. This technique enables earlier diagnosis, improves clinical management, and provides better counseling for affected families.

## Introduction

A rare disease is defined, according to Orphanet, as a condition with a prevalence of fewer than 1 in 2000 people (< 5 per 10000). More than 7000 such entities have been described; collectively, they affect about 6–8 % of the global population (roughly 300 million individuals). Multicentre studies indicate that the mean time to achieve an accurate diagnosis is approximately 6.18 years [1].

Approximately 80 % of rare diseases have a genetic origin [2, 3, 4, 5]. Within the different genetic studies used to diagnose these diseases, array comparative genomic hybridization (aCGH) has experienced significant growth over the last years.

Array CGH is a technique of molecular cytogenetics that makes it possible to detect copy number variation (CNV), deletions, and duplications throughout the entire genome with high resolution and speed compared to a control sample [6]. The samples are marked with different fluorochromes on a solid support (array) containing DNA fragments with known sequences (probes). The DNA from the patient and the control DNA compete to bind with the probes, and the color of the fluorescence provides information on the relative amount of each DNA, which makes it possible to determine the presence of gains or losses in specific regions of the genome [7]. The probes may be fragments of sequences extracted from artificial bacterial chromosomes or oligonucleotides [8].

Recently, the effectiveness and efficacy of aCGH as a first-line test in genetic diagnosis have been proven when copy number variation is suspected [9], and it is the primary genetic test for patients with autism spectrum disorder, global developmental delay, attention deficit and hyperactivity disorder, intellectual disability, and congenital malformations [10, 11, 12, 13, 14, 15, 16].

However, the criteria for administering array tests are unclear in rare diseases because their diagnostic yield for such a broad and heterogeneous group of diseases is still unknown.

Achieving a timely molecular diagnosis yields significant clinical benefits: it enables precision therapies or enrolment in clinical trials, informs prognosis and guides evidence-based surveillance for treatable complications, avoids unnecessary additional testing, and provides families with accurate counselling on recurrence risk, ultimately leading to better outcomes and lower healthcare costs.

The authors present a series of 600 array tests conducted in a pediatric unit for advanced diagnosis of rare diseases with the aim of analyzing the diagnostic value of this test, its usefulness, and yield, depending on the different clinical symptoms.

## Material and methods

An observational and descriptive study was conducted to analyze 600 aCGH tests from pediatric patients in which a rare disease was suspected, including some segregation studies, and referred to the regional Castilla y León reference Unit for Rare Diseases (DiERCyL) from any pediatric subspecialty department from January 2018 to December 2022.

The authors define a rare disease in this study as a condition with a prevalence of fewer than 1 in 2000 individuals (< 5 per 10,000), in accordance with Orphanet and European Union Regulation (EC) No 141/2000; this threshold guided case selection and data analysis.

DNA extraction was performed from peripheral blood samples collected by venipuncture with the MagNaPure Compact system (Roche Diagnostics). The concentration and quality of the samples were assessed with spectrometry.

Resolution was tailored to the clinical indication; custom higher-density arrays were used for regions of interest.

Variants were classified as pathogenic, likely pathogenic, VUS, or benign according to ACMG/AMP guidelines [17]. VUS were deemed ‘conclusive’ only when segregation and phenotype fit strongly supported causality; all other VUS were considered non-conclusive. Their impact on the symptoms of each patient was assessed by analyzing the available information from clinical-genetic databases such as Decipher (https://www.deciphergenomics.org/), UCSC (https://genome.ucsc.edu/index.html), OMIM (http://www.omim.org), or ClinVar (https://www.ncbi.nlm.nih.gov/clinvar/), among others. In some cases, a parental segregation study for the detected variants was conducted using the same technique or alternative methods such as qPCR.

In total, 40 variables from each patient were analyzed and divided into sociodemographic variables, clinical variables, and variables associated with the results from the array tests (chromosome, deletion/duplication, coordinates, size, and pathogenicity). The information was extracted from the review of clinical records provided by the doctors who requested the studies.

Before the study, informed consent for genetic testing was obtained from the patients or their guardians, as required. The Drug Research Ethics Committee of the Health District approved the study with the code PI-2023–01–1196-TFG. Statistical analyses were conducted with IBM SPSS Statistics v. 26, and an alpha risk of 0.05 was established as the limit for statistical significance. Proportional trend across ordered phenotype groups was assessed with a Cochran–Armitage test.

## Results

Out of the 600 patients analyzed initially, 57 did not present final results, and finally, 543 patients were studied. 66.3 % were male. The median age was 4.7 years (IQR: 6.36 years).

The pediatric specialty from which most children had been referred for aCGH was Pediatric Neurology, with 84.3 % of the total. The most common clinical manifestations among the children who underwent the study were ASD (38.6 %), facial dysmorphia (28.2 %), global developmental delay (27.1 %), and intellectual disability (21.0 %).

From the total of aCGH tests analyzed, 165 (30.4 %) presented variants: 98 were variants of uncertain significance (18.1 %), and 67 were pathogenic variants (12.3 %).

The array test was conclusive in 12.1 % (66/543) of the cases. Among conclusive results, 90.9 % (60/66) were pathogenic/likely pathogenic CNVs, and 9.1 % (6/66) were VUS meeting the pre-specified criteria—all de novo with strong genotype–phenotype concordance. A small number of additional CNVs were interpreted as incidental (e.g., carrier findings in autosomal-recessive disease genes) and were therefore not counted as conclusive.

Within the group of aCGH tests classified as conclusive, the median age was 3.92 years (IQR: 6.30 years). The oldest patient was 17.71 years old, and the youngest patient was one month old. No statistically significant differences were observed regarding sex for obtaining conclusive results.

Concerning the type of CNV in the conclusive arrays, 64.1 % were deletions, whereas 35.9 % were duplications. The chromosomes that presented CNVs more frequently were 15 and 16, with 14.1 % of the cases in each.

The specialties with the highest percentage of conclusive results were dysmorphology and endocrinology, with 14.3 % each, and neurology, with 12.4 % of the cases. Within the group of conclusive results, 86.4 % correspond to neurology and 6.1 % to dysmorphology.

Alterations in coordination, learning disorders, microcephaly, and epilepsy were the clinical manifestations with the highest diagnostic yield for aCGH (over 20 %) (Table 1).

**Table 1:** Conclusive results according to clinical characteristics. (GDD, Global developmental delay; ADHD, Attention deficit hyperactivity disorder; ASD, Autism spectrum disorder; Malformation, major non-facial structural defect. Facial dysmorphia (minor abnormal craniofacial features). Categories are not mutually exclusive.

	Total No.	Conclusive result ( %)
**Alterations in coordination**	14	5 (35.7 %)
**Learning disorder**	7	2 (28.6 %)
**Microcephaly**	53	12 (22.6 %)
**Epilepsy**	29	6 (20.7 %)
**GDD**	147	27 (18.4 %)
**Facial dysmorphia**	153	28 (18.3 %)
**Language delay**	48	8 (16.7 %)
**Altered phenotype**	209	32 (15.3 %)
**Intellectual disability**	114	16 (14.0 %)
**Other alterations**	15	2 (13.3 %)
**ADHD**	54	7 (13.0 %)
**Malformations**	43	6 (11.6 %)
**Macrocephaly**	21	2 (9.5 %)
**Other neurological alterations**	34	3 (8.8 %)
**ASD**	209	17 (7.7 %)
**Behavioral disorders**	14	1 (7.1 %)
**Macrosomia**	4	0 (0 %)

As shown in Table 2, diagnostic yield increased step-wise from 8.4 % in patients presenting a single clinical phenotype to 31.6 % in those with four or more phenotypes (*p* < 0.001). Among the 227 individuals with an isolated phenotype, the yield varied markedly: intellectual disability 15.8 % (3/19), autism spectrum disorder 8.1 % (8/99), global developmental delay 12.5 % (3/24), and other categories under 9 %. Combined, single-phenotype cases accounted for 28.8 % of conclusive aCGH results.

**Table 2:** Diagnostic yield of array comparative genomic hybridization (aCGH) by number of clinical phenotypes.

No. of clinical phenotypes	Total No.	Conclusive result ( %)
**1**	227	19 (8.4 %)
**2**	207	23 (11.1 %)
**3**	90	19 (21.1 %)
**≥ 4**	19	6 (31.6 %)

With those aCGH conclusive results, 49 different diagnoses were obtained, all for rare diseases, although the prevalence of 15 of these diseases is unknown. The most common disease was 15q11q13 microduplication syndrome, which was diagnosed in 5 children in the study.

## Discussion

The diagnostic yield of aCGH is 12.1 %, which shows that patients with a higher number of alterations are more likely to show alterations in the arrays.

Although the present study did not prospectively assess downstream clinical impact, molecularly confirmed diagnoses allow follow-up to be aligned with international recommendations [18, 19, 20, 21], facilitate enrolment in clinical trials, and (when no targeted intervention is available) provide families with more precise prognostic and reproductive information.

The present analysis demonstrates a dose-response relationship between phenotypic complexity and diagnostic yield: aCGH increased from 8.3 % in patients with a single phenotype to 31.6 % in those with four or more phenotypes (Table 2). Nevertheless, even with a single phenotype, the yield is clinically meaningful (15.7 % for isolated intellectual disability, 12.5 % Global developmental delay, and 8 % autism spectrum disorder), supporting the use of aCGH in minimal presentations. A potential referral bias should be acknowledged: more complex cases are probably referred for aCGH more frequently, which could magnify the observed gradient.

Globally, an estimated ∼300 million individuals — and ∼30 million across Europe — are living with a rare disease, underscoring the international relevance of early genomic diagnosis and equitable access to aCGH [1,22]. Although this study applied the Orphanet threshold for rare diseases (< 1 in 2000 individuals), other jurisdictions—such as the FDA, which uses < 200,000 affected individuals in the United States- employ different cut-offs; this variability should be considered when comparing diagnostic yields across studies.

It is estimated that 80 % of all rare diseases have a genetic origin, which explains the importance of genetic studies for diagnosis and research into these diseases. Also, genetics is an essential tool to counsel families with patients affected by rare diseases [2, 3, 4, 5].

The aCGH tests are genetic tests that make it possible to identify the presence of CNV rapidly and effectively. Their use is increasingly common, but the diagnostic yield in such a heterogeneous group of diseases is still unknown.

This study analyzes a series of 543 patients with a suspected rare disease who underwent aCGH. One of the advantages of this technique is that it does not require diagnostic guidance, unlike gene panel testing, and this makes it particularly interesting for young infants, who may not have yet expressed some of the clinical manifestations of their condition. In this series, the median age was 3.92 years. No other studies have been found in the literature that analyze the age of the patients with conclusive results, but the mean time until the diagnosis of a rare disease in Spain is known to be 6.18 years [1]. Reaching an early diagnosis for these diseases has a significant impact. On the one hand, the identification of conditions such as AHDH, ASD, or intellectual disability makes it possible to guide an early intervention, which could improve the prognosis of the affected children [23]. On the other hand, a diagnosis represents the end of a long series of consultations and tests for the families of those affected [24].

The total diagnostic yield of the arrays in the present sample is 12.3 %, similar to what has been reported in most of the studies that analyze aCGH series in specific disorders in which it is indicated (10–15 %] [9,10,23]. However, from a clinical perspective and in the context of rare diseases, it is more important to analyze the yield of the study concerning the clinical manifestations that justified their use. The present study shows that the clinical characteristics with the highest percentage of conclusive results are alterations in coordination at 35.7 % and learning disorders at 28.6 %. No previous studies in the literature have analyzed the diagnostic yield of arrays in these disorders separately. The yield for some of the most common clinical manifestations is similar to what has been reported by other authors: altered phenotype (15.3 %), intellectual disability (14 %), and ASD (7.7 %) [9,13,23,25].

This study also shows that diagnostic yield increases with phenotypic complexity, rising from 8.4 % when a single clinical phenotype is present to 31.6 % in patients who display four or more concurrent phenotypes (Table 2). This finding is in line with what was described in other studies [9].

The present results confirm the effectiveness and efficacy of the arrays for diagnosing pediatric rare diseases and establish the differences related to the different clinical manifestations. The presence of more than one clinical alteration significantly increases the diagnostic yield of the test. When a rare disease is suspected, the early implementation of array tests can shorten the time until diagnosis. This makes it possible to provide better management of the conditions and adequate genetic counseling for the families.

## Declaration of generative AI and AI-assisted technologies

During the preparation of this work, the authors used ChatGPT (OpenAI) solely for language editing (grammar, clarity, and style). After using this tool, the authors reviewed and edited the content as needed and take full responsibility for the content of the publication. No identifiable patient data were entered into the tool.

## Funding

The authors received no financial support for the research, authorship, and/or publication of this article.

## Conflicts of interest

The authors declare no conflicts of interest.
